# Long-term effects and costs of pelvic floor muscle training for prolapse: trial follow-up record-linkage study

**DOI:** 10.1007/s00192-022-05272-9

**Published:** 2022-06-28

**Authors:** Linda Fenocchi, Catherine Best, Helen Mason, Andrew Elders, Suzanne Hagen, Margaret Maxwell

**Affiliations:** 1 Yunus Centre for Social Business & Health, M201 George Moore Building, Glasgow Caledonian University, Cowcaddens Road, Glasgow, G4 0BA UK; 2 Nursing, Midwifery and Allied Health Professions Research Unit, University of Stirling, Stirling, UK; 3 Nursing, Midwifery and Allied Health Professions Research Unit, Glasgow Caledonian University, Glasgow, UK

**Keywords:** Pelvic organ prolapse, Record linkage, Longitudinal cost analysis, Pelvic floor muscle training, Randomized controlled trial, Long-term follow-up

## Abstract

**Introduction and hypothesis:**

Pelvic organ prolapse affects around 40% of women aged over 50 years. A multicentre parallel group randomised trial (the Pelvic Organ Prolapse PhysiotherapY (POPPY) trial) demonstrated that pelvic floor muscle training (PFMT) was effective in reducing prolapse symptoms compared with no treatment. However, insight into the long-term impact of PFMT on health outcomes and health-service utilisation is scarce.

**Methods:**

This study utilised linkage of Scottish administrative health records to follow-up POPPY trial participants resident in Scotland over 11 years. Mixed effects logistic regression determined the likelihood of receiving further prolapse treatment for those in the PFMT and control groups. Analyses were adjusted for age group, prolapse stage, baseline symptom severity and attitude towards surgery. A cost assessment estimated longitudinal costs to the UK National Health Service (in Scotland) of accessing further prolapse treatment for each trial group.

**Results:**

Two hundred and ninety-three women, aged 25 to 79 years, were followed up. One hundred and forty-one women (48.1%) had received further prolapse treatment: 65 (of 149; 43.6%) in the PFMT group compared with 76 (of 144; 52.8%) in the control group. PFMT was associated with a reduction in the odds of any prolapse treatment during follow-up (AOR 0.61; 95% CI 0.37 to 0.99). Total cost of secondary care was £154,544 (GBP) in the PFMT group and £172,549 (GBP) in the control group.

**Conclusions:**

Although PFMT did not lead to significant differences in total costs for further prolapse treatment over a post-intervention period of more than 10 years, it reduced the overall long-term risk of requiring hospital-based treatment for pelvic floor disorders.

## Introduction

Pelvic organ prolapse (POP) is the loss of support for the uterus, bladder or bowel that results in the descent of one or more of these organs into the vagina [1]. Prolapse affects around 40% of women aged over 50 years [2]. Risk factors include pregnancy, childbirth, and ageing. The symptoms of POP can affect daily activities and quality of life [3], and women may experience pelvic pain, bladder and bowel dysfunction. The most common treatments for POP in women include surgery, vaginal support pessary and pelvic floor muscle training (PFMT). Women often access PFMT first as a conservative (nonsurgical) measure [4]. PFMT is an effective (majority level 1 evidence [1]) non-surgical option that has been shown to improve prolapse symptoms and health-related quality of life for women experiencing POP [5–7]. PFMT is aimed at improving the function of the pelvic floor muscles to increase the structural support for the pelvic organs. This is achieved by targeted exercises to strengthen and improve coordination of the muscles.

Economic analyses are an important basis for determining the cost-effectiveness of different intervention options that might be used. Yet there are limited data on the cost-effectiveness of conservative interventions for POP [8, 9]. Previous modelling analysis of costs and benefits of non-surgical and surgical treatments for POP included only pessary as a non-surgical intervention [10]. Efforts to establish the economics of PFMT have been conducted within the scope of trials. A systematic review of PFMT for stress urinary incontinence in women identified only one economic evaluation [11]. It identified a cost-utility analysis of PFMT delivered by a mobile phone app, which concluded that the app treatment was cost-effective, estimated at €7,615.50 (Euro) per quality-adjusted life year (QALY) [12]. However, the trial that it related to had not collected data on the presence of co-existing POP in the participants [13]. The cost-effectiveness of PFMT, in comparison with watchful waiting, has been assessed over a 2-year follow-up period in a patient population with symptomatic mild POP [14]. Although the study reported a positive effect of PFMT on POP symptoms, and estimated an incremental cost-effectiveness ratio of €31,983 (Euro; 95% CI: 76,652 to 88,078) per QALY, it noted that direct medical costs per person were reasonable and suggested that decisions should be based on clinical need and patient preference. Economic findings of the Pelvic Floor Muscle Training for Secondary Prevention of Pelvic Organ Prolapse (PREVPROL) trial suggested a cost per QALY range from £21,996 to £29,409 (GBP) [15]. However, the effectiveness and cost-effectiveness evaluations of PFMT have, to date, been limited to relatively short follow-up periods. The economic impact of PFMT is likely to occur over years as opposed to months.

Evidence of the impact of PFMT on women’s longer-term treatment outcomes is scarce. The Pelvic Organ Prolapse PhysiotherapY (POPPY) trial was a large, pragmatic trial of PFMT for prolapse (registration number NCT00476892), which concluded that PFMT was effective in reducing reported prolapse symptom severity at 12-month follow-up in treatment-naive women presenting with stage 1 to 3 prolapse [16]. The net cost of delivering PFMT during the trial was reported to be £130 (GBP) per woman, but conclusions regarding the potential of PFMT to be a cost-effective treatment were limited by uncertainty about whether PFMT changed the need for subsequent treatment in the longer term or delayed it. Investment in Scotland in health record linkage makes it possible to follow-up original POPPY trial participants using record linkage of hospital admissions and outpatient datasets.

To understand if women who undertook PFMT were less likely to receive further secondary care treatment related to their prolapse during the follow-up period than women who were in the control condition, routinely collected administrative data were used to investigate health service use over a follow-up period of up to 11 years in the 310 original POPPY trial participants resident in Scotland. This accounted for 69.3% of the 447 participants in the original trial cohort. The aim of the study was to investigate longer term associations between PFMT and the need for prolapse intervention; the time to prolapse intervention; and the long-term costs associated with accessing further prolapse treatment.

## Materials and methods

### Record linkage

Estimates of long-term effects and costs associated with accessing further prolapse treatment over time required linkage to routinely collected administrative health care data about individual patients, specifically Scottish Morbidity Records [17] for outpatients (SMR-00) and inpatients and day cases (SMR-01), for participants in the original POPPY trial who were based in Scotland. Approval for linkage was obtained from the National Health Service (NHS) Scotland Public Benefit and Privacy Panel for Health and Social Care. Following approval, NHS National Services Scotland’s Electronic Data Research and Innovation Service (eDRIS, now part of Public Health Scotland, www.isdscotland.org/products-and-services/edris/use-of-the-national-safe-haven) undertook the linkage of the POPPY trial data with the SMR-00 and SMR-01 datasets and information on deaths from National Records Scotland. Anonymised linked data files were made available for analysis via the National Safe Haven.

### Analysis dataset

Longitudinal health care data were available for a post-intervention period of 10 years and 9 months (September 2007 to May 2018). The primary outcome measure was the occurrence of any related secondary care treatment for pelvic floor disorders during follow-up, defined as Office of Population Censuses and Surveys Classification of Interventions and Procedures version 4 (OPCS4) procedure code P18, P22–P24, P26, Q07–Q08, Q54, M51–M58, A70 or H57 as the main operation or other operation and/or International Statistical Classification of Diseases and Related Health Problems, 10th revision (ICD10) diagnosis code N81, N99.3, N39.3, N39.4, R32, K62.2, K62.3 or R15 as main or other condition [18, 19]. The primary outcome was expressed as a binary variable.

### Statistical analysis

Analysis was conducted in Stata version 14. Mixed effects logistic regression was used to estimate effect size for the association between PFMT and any related secondary care treatment for pelvic floor disorders (odds ratios [OR] and 95% confidence intervals [CI]). Analyses were adjusted for the variables as used in the original POPPY trial analysis (baseline symptom severity [Pelvic Organ Prolapse Symptom Score, POP-SS], score range 0–28, higher scores indicate worse severity [20]; prolapse stage [Pelvic Organ Prolapse Quantification, POP-Q]; stages 1–3 included in the trial, higher stages indicate worse severity [21]; and attitude to prolapse surgery). Attitude towards surgery was assessed with the question “If you were to be offered surgery in the future as a treatment for your prolapse, how would you feel about it?” with response options ‘I would like to avoid surgery if at all possible”, “I am willing to have surgery but only if it is unavoidable” and “I am keen to have surgery”. In addition, treatment centre was included in the model as a random effect. Age group was included as an additional covariate, as over an 11-year follow-up period, the impact of age may be increased. Missing prolapse symptom severity scores were imputed at median because of the relatively small sample size. Results for a complete case analysis are presented as a sensitivity analysis to check the effects of this approach to missing data. As a secondary analysis, Cox regression was used to analyse time-to-event data. That is, time from the intervention to any related secondary care treatment for pelvic floor disorders was compared between treatment and control conditions. The analysis was stratified by attitude to surgery and was adjusted for symptom severity at baseline, prolapse stage, age group and body mass index (BMI).

### Resource utilisation and cost estimation

Resource use data for the predefined OPCS4 main operation codes for pelvic floor disorders (P18, P22–P24, P26, Q07–Q08, Q54, M51–M58, A70, H57) were identified. Records were excluded where a “did not attend” code was present. A simple mapping of predefined OPCS4 main operation codes of interest to Healthcare Resource Groups (HRG) and thereafter to the relevant currency codes was used to extract unit cost data from NHS reference costs [18, 22]. Table 1 provides a summary of the reference costs for each procedure present in the dataset (P22, P23, P24, Q07, Q08, M53). Information about community-based treatment (for example, GP appointments or drug treatment) was not available and estimated costs were not included. Estimates of health care utilisation were costed for F2 Gynaecology outpatient (SMR-00) and inpatient (SMR-01) episodes of care. Excess bed days were calculated as the difference between the number of bed days recorded and the average number of bed days for POPPY participants with the same main operation code. The administrative data were assessed for differences in resource use between control and intervention groups, including the influence of women who required more than one episode of care during the time of follow-up on cost estimates. Information is presented in aggregate to meet the data protection requirements of NHS National Services Scotland’s Statistical Disclosure Control Protocol [23]. In line with the original POPPY trial costs base year of 2009, reporting of economic analysis was in 2009 UK pound sterling (GBP £; GDP deflators [24]).

**Table 1:** Unit costs applied to SMR-00 and SMR-01 data, 2017–2018 prices (GBP)

OCPS-4 codes	HRG description	Currency codes	National average unit costs (GBP)		
			Day case (GBP)	Elective inpatient (GBP)	Elective inpatient excess bed days (GBP)
P26—outpatient	Insertion/removal supporting pessary	MA23Z	183.52		
M533, M534, M536	Vaginal tape operations for urinary incontinence	LB51B	1,527	2,019	574
P241, P242, P243, P245, P246, Q071, Q072, Q073, Q081, Q082, Q083, Q547	Complex, laparoscopic or endoscopic, upper genital tract procedures	MA28Z	2,649	4,540	641
P221, P222, P223, P228, P229, P235, P244, P247, P248, P249	Major open lower genital tract procedures	MA03D	1,630	2,974	474
P231, P236, P237	Very major open, upper or lower genital tract procedures	MA02C	1,811	4,663	525
M531, M535, M537, P234, P238, P239	Intermediate open lower genital tract procedures	MA04D	1,400	2,450	547
P232, P233	Major open lower genital tract procedures	MA03D	1,630	2,974	474
Q074, Q075, Q076, Q078, Q079, Q088, Q089, Q544, Q545, Q546	Very major open, upper or lower genital tract procedures	MA02C	1,811	4,663	525
Q541, Q542, Q543, Q548	Intermediate, laparoscopic or endoscopic, upper genital tract procedures	MA09B	1,819	2,748	288
M532, M538, M539	Minor lower genital tract procedures	MA22Z	1,148	1,817	–

Currency units (average for the year 2017) per 1 GBP: = 1.14615 EUR; =1.282692 USD; = 143.837608 JPY; = 1.263125 CHF; = 1.663342 CAD; = 1.6733 AUD; = 1.799358 NZD; = 17.011558 ZAR; = 24.309267 MXN; = 4.7113 AED. Source (last accessed online 11 April 2022): https://www.gov.uk/government/publications/exchange-rates-for-customs-and-vat-yearly]

Source: National Schedule of Reference Costs—Year 2017–2018—NHS trust and NHS foundation trusts

*HRG* Healthcare Resource Group, *SMR* Scottish Morbidity Records, *OPCS* Office of Population Censuses and Surveys Classification of Interventions and Procedures version 4

## Results

### Participants

Linked follow-up data were available for 293 of the 447 participants (65.5%) in the original POPPY trial. The characteristics of participants included in the analysis are summarised in Tables 2 and 3, using data measured at baseline in the original POPPY trial. The distribution of characteristics is similar to that of the full POPPY trial sample. During the follow-up period, there were 15 participants (5%) who died. The all-cause mortality rate was 5.4% in the intervention group and 4.9% in the control group. All other outcome measures in this analysis used the hospital episode activity data in the linked dataset, although all episodes with a pelvic floor disorder were identifiable from OPCS4 procedure codes alone (there were no cases identified from ICD10 diagnosis codes alone).

**Table 2:** Baseline characteristics of participants available for follow-up: categorical variables

Variable	Value	Intervention, PFMT (%)	Control (%)	Total (%)
Attitude to surgery	Would like to avoid surgery if possible	18 (12.1)	15 (10.4)	33 (11.3)
	Willing to have surgery if unavoidable	131 (87.9)	128 (89.6)	260 (88.7)
Stage of prolapse (POP-Q)	1	18 (12.1)	17 (11.8)	35 (12.0)
	2	107 (71.8)	108 (75.0)	215 (73.4)
	3	24 (16.1)	19 (13.2)	43 (14.7)
Age group	25–44	20 (13.4)	23 (16.0)	43 (14.7)
	45–64	92 (61.7)	91 (63.2)	183 (62.5)
	65–79	37 (24.8)	30 (20.8)	67 (22.9)
Parity	0–1	19 (12.8)	15 (10.4)	34 (11.6)
	2	70 (47.0)	59 (41.0)	129 (44.0)
	3	41 (27.5)	49 (34.0)	90 (30.7)
	4	17 (11.4)	17 (11.8)	34 (11.6)
	Missing	2 (0.7)	4 (2.8)	6 (2.1)

*PFMT* pelvic floor muscle training, *POP-Q* Pelvic Organ Prolapse Quantification

**Table 3:** Baseline characteristics of participants available for follow-up: continuous variables

Variable	Statistic	Intervention (PFMT)	Control	Total
BMI	*n*	144	135	279
	Mean	27.3	27.2	
	Standard deviation	4.9	4.4	
Months of bother with prolapse symptoms	*n*	131	128	259
	Mean	22.4	22.0	
	Standard deviation	33.4	35.9	
Prolapse symptom severity (POP-SS)	*n*	142	135	277
	Mean	10.5	10.1	
	Standard deviation	6.0	5.8	

*BMI* body mass index, *PFMT* pelvic floor muscle training, *POP-SS* Pelvic Organ Prolapse Symptom Score

### Women receiving treatment during follow-up

The overall proportion of women receiving any secondary care treatment for pelvic floor disorders was 48.1% (141 out of 293), with the rate being 43.6% (65 out of 149) in the intervention group compared with 52.8% (76 out of 144) in the control group. This corresponds to an absolute risk reduction of 9% and a number needed to treat of 11. That is, 11 women would need to receive the PFMT intervention in order to prevent one further treatment during follow-up.

The mixed effects logistic regression on “any treatment during follow-up” (primary outcome measure) with adjustment for baseline prolapse symptom severity, motivation for prolapse surgery, age group and prolapse stage, with random effect of centre, estimates a significant treatment effect (odds ratio 0.61, 95% CI 0.37 to 0.99, p=0.047). The estimated parameters from the model are shown on Table 4.

**Table 4:** Mixed effects logistic regression on any treatment during follow-up with random effect of centre

Variable	OR (95% CI); POP-SS imputed at median; *n*=293	OR (95% CI); complete cases *n*=277	*p* value for imputed
Intervention (PFMT vs control)	0.61 (0.37 to 0.99)	0.59 (0.36 to 0.98)	**0.047**
Prolapse symptom severity	1.05 (1.00 to 1.09)	1.05 (1.00 to 1.10)	**0.037**
Attitude to surgery	0.53 (0.24 to 1.19)	0.53 (0.23 to 1.21)	0.126
Stage of prolapse 2 (reference category stage 1)	2.03 (0.90 to 4.66)	1.89 (0.82 to 4.35)	0.091
Stage of prolapse 3 (reference category stage 1)	3.00 (1.07 to 8.41)	2.84 (1.00 to 8.07)	**0.036**
Age band 45–64 (reference category <45)	1.82 (0.86 to 3.86)	1.95 (0.90 to 4.22)	0.116
Age band 65–79 (reference category <45)	4.62 (1.90 to 11.36)	4.99 (1.97 to 12.62)	**0.001**

Statistically significant, *p≤*0.05, in bold

*CI* confidence interval, *OR* odds ratio, *PNFT* pelvic floor muscle training, *POP-SS* Pelvic Organ Prolapse Symptom Score

The likelihood ratio test for the mixed effects logistic regression model versus a model with fixed effects only (likelihood ratio test Chi-squared=2.74, *p*=0.0489) indicated that the random effect of centre is required. The PFMT intervention is associated with a reduction in the odds of any treatment during follow-up. The adjusted odds ratio of 0.61 corresponds to a relative risk reduction of 0.80, indicating that the probability of need for further treatment is 20% lower in the group receiving the PFMT intervention than in the control condition after adjustment for covariates.

### Analysis of time to first treatment

**Fig. 1:**
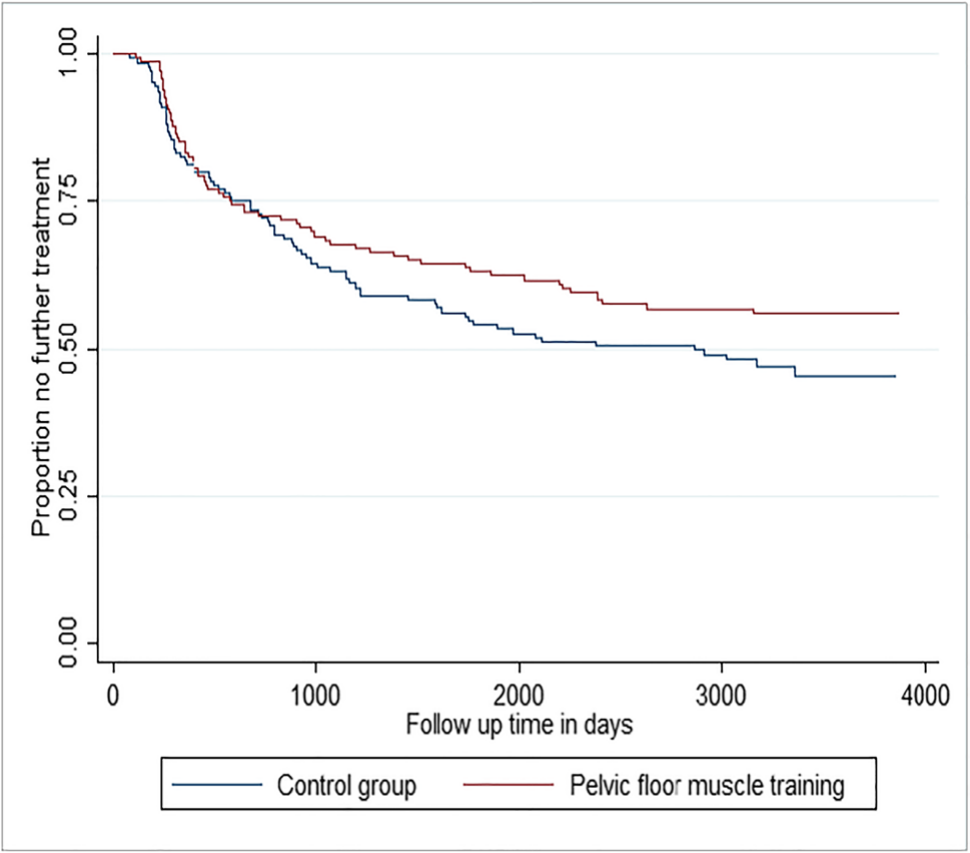
Kaplan–Meier survival estimates by intervention status

This study examined whether there was any difference between the PFMT and control groups in the time until they received pelvic floor disorder secondary care treatment during follow-up. This was in order to determine whether PFMT was effective in delaying any further pelvic floor disorder treatment. First, the overall follow-up periods of the PFMT and control groups were compared. The time in follow-up of the two groups (i.e. the time at risk and under observation) was found to be comparable: (mean time in follow-up in the intervention group was 3,415.3 days (SD=262.5) and the mean in the control group was 3,403.0 days (SD=267.9)). The survival function for the PFMT and control group is shown in Fig. 1. The survival function shows how the proportion of women in the two groups who have not received any further treatment decays over time.

Cox regression (Table 5) was used to test whether women in the control group were likely to receive treatment sooner than women in the PFMT group. The Cox regression included 264 women and 125 events. This model estimated a hazard ratio in favour of the intervention group of 0.65 (95% CI 0.46 to 0.94), *p*=0.020. This indicates that the intervention did delay the need for further treatment and that during follow-up women in the PFMT group had a 35% reduction in hazard for treatment.

**Table 5:** Cox regression on time to any treatment

Variable	Hazard ratio (95% confidence interval)	*p*
Intervention	0.65 (0.46 to 0.94)	**0.020**
Prolapse symptom severity score baseline (missing imputed at median)	1.06 (1.02 to 1.09)	**0.001**
Stage of prolapse 2 (reference category stage 1)	1.48 (0.77 to 2.85)	0.245
Stage of prolapse 3 (reference category stage 1)	2.40 (1.13 to 5.13)	**0.023**
Age band 45–64 (reference category <45)	1.66 (0.88 to 3.10)	0.115
Age band 65–79 (reference category <45)	2.58 (1.29 to 5.17)	**0.008**
BMI	0.95 (0.91 to 0.99)	**0.014**

Statistically significant, *p*≤0.05, in bold

*BMI* body mass index

### Economic assessment

For the economic assessment, follow-up data covering the period 2007 to 2018 were available for 293 women involved in the POPPY trial for F2 Gynaecology outpatient (SMR-00) and inpatient (SMR-01) episodes of care. A large number of outpatient records (84%, *n*=1,193) recorded no data on the main condition or procedure code, preventing identification of whether the appointments were, or were not, related to pelvic floor disorder. Using procedure codes of interest, 111 SMR-00 outpatient episodes of care (note: not equivalent to individuals) were identified and resource use costs were estimated. Outpatient resource use by both groups was broadly similar, with 59 (control group) and 52 (PFMT group) episodes of care recorded. POPPY participants' health care utilisation costs for procedures of interest (F2 Gynaecology), 2007 to 2018 (GBP 2017–2018 prices) SMR-00: outpatient, was £18,740 for the PFMT group and £7,227 for the control group. For SMR-01 inpatient 109 episodes of care were identified using procedure codes of interest, involving 94 (32%) women: 50 in the PFMT group; 59 in the control group (Table 6). The majority of procedures coded within the episodes of care were for colporrhaphy/vaginal wall repair (P22, P23, P24, Q54). (Note that some patients will have received more than one procedure of interest during a single health care episode.) Owing to issues of confidentiality, SMR-01 data cannot be broken down further. Aggregate costs associated with inpatient treatment received for POP were estimated as £163,267 (PFMT group) and £184,748 (control group) (GBP 2017–2018 prices), inclusive of excess bed days. The difference is potentially an artefact of price deflation adjustment.

**Table 6:** Summary of Scottish Morbidity Records (SMR)-01 inpatient episodes of care and related costs (2007–2018), by trial group

	Intervention	Control	Total
Women, *n*	46	48	94
Episodes of care (code of interest), *n*	50	59	109
Total resource use (excluding excess bed days) (GBP)	154,544	172,459	327,003
Average cost per episode of care (excluding excess bed days; GBP)	3,360	3,593	3,479
Women with more than one episode of care, *n*	Withheld	Withheld	11
Proportion of episodes of care accounted for by women receiving more than one, % (number of episodes)	16 (8)	31 (18)	24 (26)
Average cost per woman, one episode of care (GBP)	3,077	2,797	2,937
Average cost per woman, more than one episode of care (GBP)	6,331	8,252	7,292

Information is presented in aggregate to meet the data protection requirements of the NHS National Services Scotland’s Statistical Disclosure Control Protocol Version 3.0. (2015). (Accessed 4 March 2019 https://www.isdscotland.org/About-ISD/Confidentiality/disclosure_protocol_v3.pdf). Small numbers have been necessarily withheld

Intervention (PFMT); control (lifestyle advice leaflet); GBP, 2017–2018 prices deflated to 2009 UK pound sterling

Predefined Office of Population Censuses and Surveys Classification of Interventions and Procedures version 4 main operation codes of interest: P18, P22–P24, P26, Q07–Q08, Q54, M51–M58, A70, H57

*PFMT* pelvic floor muscle training

When costs were estimated with and without women who required more than one episode of care during the follow-up period, 11 women had more than one episode of care during the follow-up period and accounted for 24% of all surgical interventions observed. Table 6 indicates that average cost per woman with one episode of care was £2,973 (£3,077 in the PFMT group and £2,797 in the control group). Average cost diverged when considering women with more than one episode of care: £6,331 for the PFMT group and £8,252 for the control group. Analysis of POPPY trial participants’ outpatient and inpatient health care utilisation costs for procedures of interest (F2 gynaecology) did not find an observed difference between the PFMT group and the control group at 11 years post-PFMT intervention.

## Discussion

The longitudinal data record linkage study examined the long-term impact of PFMT on health outcomes and health service utilisation of POPPY trial participants in Scotland, UK. The record linkage study, which focused on individual women’s treatment, provides evidence that PFMT reduces the overall long-term risk of requiring hospital treatment for pelvic floor disorders, over a post-intervention period of more than 10 years. There is also evidence that PFMT extends the time for which hospital treatment is not required. This effect was evident only after adjustment for age suggesting that age is an important predictor of long-term treatment. Older women (over 65 years of age) were more likely to have further treatment and to receive it sooner independent of treatment allocation. Economic interpretation of longitudinal follow-up data for POPPY participants did not indicate a difference in the use of inpatient health care resources between the groups. In an effort to assess potential distortion of resource use cost assessment owing to the small numbers of participants, costs were estimated with and without women who required more than one episode of care during the time of follow-up. Although this indicated that the intervention group had fewer and less costly repeat episodes, small numbers mean that these findings must be treated cautiously. On the basis of the follow-up data obtained from the administrative data sets, it was concluded that there was no clear difference in resource use costs between the intervention group and control group participants at 11 years post-PFMT.

Cost estimates for longitudinal follow-up of POPPY participants were based on the main operation code. Some participants received more than one procedure of interest during a single health care episode. This has implications for the confidence that can be placed on resource estimations because the average costs cannot reflect the influences of other procedures and personal characteristics such as other diagnoses and the presence of co-morbidities that affect the resulting costs per patient. It was not feasible to gather information about post-operative care, such as medications, general practitioner or practice nurse support.

The main limitation of our study was the exclusion of records in the Scottish Morbidity Records for outpatient and inpatient datasets owing to the inability to determine whether health care resource use was relevant because of missing main condition or procedure codes recorded. The likely implication of this is that resource use costs are underestimated. This was particularly apparent for SMR-00 outpatient records because procedure codes were recorded in only 16% of records and no main condition was recorded in any of the records. An operation code is non-mandatory for this dataset, preventing the search of only episodes relevant to pelvic floor disorders. It also affects the estimation of costs for POPPY participants related to non-surgical health care in outpatient care settings, such as the use of a vaginal support pessary, which is a main non-surgical option for the management of POP.

As the first study to assess the impact of PFMT on women’s longer-term treatment outcomes including the need for pelvic floor disorder intervention, time to intervention, and the long-term costs associated with accessing further treatment, this study provides unique information. For research, the importance of planned long-term follow-up of trial participants is highlighted. For practice, this data linkage study provides further evidence to support recommending PFMT to women as first-line treatment as PFMT may delay, or possibly avoid, the need for more treatment. Over a post-intervention period of more than 10 years, PFMT reduced the overall long-term risk of requiring hospital treatment for pelvic floor disorders. Although the total cost was broadly similar for both the intervention and the control groups, assessment of costs when patients required more than one episode of care suggested that targeted planning of care such as PFMT may be advisable for health care resource planning. The study also provides reflections on lessons learned about the benefits and limitations of using routine administrative data, adding to the limited methodological knowledge on the use of routine linked health data for longer-term follow-up.

## Declarations

### Linkage approval process

An application was made to the NHS Scotland Public Benefit and Privacy Panel (PBPP) for Health and Social Care in 2017. Following a Tier 1 review (April 2017), the application was initially referred for a Tier 2 (out-of-meeting) review but was subsequently referred to a Tier 2 panel meeting (November 2017), which required the PI to attend and respond to questions. Approval was obtained in January 2018 (PBPP Application 1617-0049/Semple: Using linked data to follow up longer-term outcomes of POPPY trial participants).
